# Comparative analysis of inflamed and non-inflamed colon biopsies reveals strong proteomic inflammation profile in patients with ulcerative colitis

**DOI:** 10.1186/1471-230X-12-76

**Published:** 2012-06-24

**Authors:** Nina Aagaard Poulsen, Vibeke Andersen, Jens Christian Møller, Hanne Søndergaard Møller, Flemming Jessen, Stig Purup, Lotte Bach Larsen

**Affiliations:** 1 Department of Food Science, Aarhus University, Tjele 8830, Denmark; 2 Medical Department, Viborg Regional Hospital, Viborg 8800, Denmark; 3 Medical Department, SHS Aabenraa, Aabenraa 6200, Denmark; 4 Institute of Regional Health Services Research, South Danish University, Odense 5000, Denmark; 5 Pathological Department, Viborg Regional Hospital, Viborg 8800, Denmark; 6 National Food Institute, Technical University of Denmark, 2800 Kgs Lyngby, Denmark; 7 Department of Animal Science, Aarhus University, Tjele 8830, Denmark

**Keywords:** Inflammatory bowel disease, Ulcerative colitis, Colon biopsies, Candidate markers, MS-based proteomics

## Abstract

**Background:**

Accurate diagnostic and monitoring tools for ulcerative colitis (UC) are missing. Our aim was to describe the proteomic profile of UC and search for markers associated with disease exacerbation. Therefore, we aimed to characterize specific proteins associated with inflamed colon mucosa from patients with acute UC using mass spectrometry-based proteomic analysis.

**Methods:**

Biopsies were sampled from rectum, sigmoid colon and left colonic flexure from twenty patients with active proctosigmoiditis and from four healthy controls for proteomics and histology. Proteomic profiles of whole colonic biopsies were characterized using 2D-gel electrophoresis, and peptide mass fingerprinting using matrix-assisted laser desorption/ionization time-of-flight mass spectrometry (MALDI-TOF MS) was applied for identification of differently expressed protein spots.

**Results:**

A total of 597 spots were annotated by image analysis and 222 of these had a statistically different protein level between inflamed and non-inflamed tissue in the patient group. Principal component analysis clearly grouped non-inflamed samples separately from the inflamed samples indicating that the proteomic signature of colon mucosa with acute UC is strong. Totally, 43 individual protein spots were identified, including proteins involved in energy metabolism (triosephosphate isomerase, glycerol-3-phosphate-dehydrogenase, alpha enolase and L-lactate dehydrogenase B-chain) and in oxidative stress (superoxide dismutase, thioredoxins and selenium binding protein).

**Conclusions:**

A distinct proteomic profile of inflamed tissue in UC patients was found. Specific proteins involved in energy metabolism and oxidative stress were identified as potential candidate markers for UC.

## Background

Ulcerative colitis (UC) is a chronic relapsing inflammatory disease of the colon. Together with Crohn´s disease (CD), UC is referred to as chronic inflammatory bowel diseases (IBD). In Denmark, the incidence of UC is approximately 10 per 100,000 [1]. The underlying pathology of the disorder is complex and far from fully understood [2-7].

High throughput technologies like microarray and proteomic approaches can be utilized to identify disease markers, which can be used for diagnostic and monitoring purpose [8-12]. Based on these technologies several candidate markers being correlated with IBD disease phenotypes have been revealed, mainly identified from serum samples [13-17], but also from colon tissues [18-22]. Recently, biopsies from IBD patients were successfully examined in a multigene analysis resulting in diagnostic precision of IBD [18]. Thus, seven marker genes were identified, where expression in colonic mucosal biopsies differed between patients with UC, patients with CD and patients with non-IBD. Moreover, by the use of these marker genes, the authors were able to distinguish between 38 patients with UC, 28 patients with CD and 20 patients with non-IBD in a prospective panel [18].

Fecal calprotectin is a very sensitive marker for intestinal inflammation, but it is not a specific marker and increased levels are also found in neoplasia, infections, polyps, and with use of non-steroidal anti-inflammatory drugs and increasing age [23]. Thus, intestinal tissue samples are suggested to be the most important source for the identification of disease markers for further validation [24].

Markers may serve a wide range of purposes in IBD, such as diagnostic purposes, providing objective measures for disease activity, and as indicators for treatment outcome. Thus, a panel of markers is needed to cover these various clinical settings. For example the symptoms of IBD are often unspecific, and diagnosis may be delayed with devastating impact on disease progression as a result [25]. Therefore, fast and reliable diagnostic tools are wanted [26].

During UC inflammation both the mucosa and submucosa will be affected, resulting in tissue damage and ulceration, and studies based on whole biopsies can thus be challenging due to tissue complexity. However, routine endoscopic evaluations enable easy examination of whole biopsies. With this in mind, proteomics is an ideal hypothesis-free approach to shed light on molecular characterization and diagnostics of intestinal inflammation [24].

The aim of this study was to apply a comparative proteomic approach in order to characterize the proteomic signature of inflamed versus non-inflamed colonic tissue from twenty UC patients in order to establish a baseline of markers, which are associated with active UC. This is the first study analysing the global proteomic signature of affected and non-affected colon tissues from more than a few UC patients using 2-dimensional gel electrophoresis (2-DGE) based proteomics coupled with mass spectrometry (MS). We found 222 protein spots which were significantly different expressed in inflamed versus non-inflamed tissue. Of these, 43 protein spots were identified and assigned to 33 individual proteins.

## Methods

### Patients and controls

Twenty patients (1:1 female–male ratio, mean age 40 (aged 18–67), 12 non-smokers, 7 former smokers and 1 current smoker) with proctosigmoiditis were recruited from Viborg Regional Hospital, Denmark. Diagnosis of proctosigmoiditis was based on clinical, endoscopic, and histological examinations [27]. Of these, four patients were diagnosed with UC for the first time at the endoscopy. Among the 20 patients, ten were treated with 5-aminosalicylic acid, (5-ASA) one with salazopyrine, one with a diuretic and renin-angiotensin inhibitor due to arterial hypertension, and eight were without daily treatment. In addition, four voluntary healthy controls (1:1 female–male ratio, mean age 32 (aged 18–50)) without any familial disposition for inflammatory bowel disease, daily medication, or any known diseases were recruited by announcement. They were all without inflammation at the endoscopy.

### Sample collection

Biopsies (3–10 mg) from patients and healthy controls were sampled by endoscopy conducted at the Medical Department at Viborg Regional Hospital, Denmark. All study subjects fasted 12 h prior to endoscopy. Samples were taken for both proteomics and histological analyses from each location. From the healthy controls replicate biopsies (two for proteomics, two for histology) were sampled from rectum (RE), sigmoid colon (SI) and left colonic flexure (LF), respectively, while, from patients, replicate biopsies (two for proteomics, two for histology) were sampled from acutely inflamed mucosa from RE (affected sample) or from non-inflamed mucosa from LF (control sample), respectively. Biopsy specimens were immediately stored in dry ice and subsequently stored at −80°C until preparation for 2-DGE.

### Histological evaluation

The histological examination of inflamed, non-inflamed and normal tissue was conducted on specimens taken in the same area as those used for proteomic profiling and fully agreed with the clinical assessment. The excised biopsies were evaluated by a trained pathologist using hematoxylin and eosin staining. The pathologist was blinded for the clinical evaluation of the patients and the proteomic results. The specimens were evaluated by conventional histological criteria, including crypt distortion, goblet cell loss, inflammation in the mucosal lamina propria, subcryptal leukocyte infiltration and absence of changes specific for other diseases such as granulomas [28,29]. Furthermore, the biopsies were graded using a simplified method for histological assessment of inflammation in UC [28], which includes crypt distortion (score 0–4), crypt inflammation (score 0–3) and subcryptal leukocyte infiltration (score 0–2) (maximum score was 7). Biopsy score from the healthy persons were 0 (25%-*7*5% percentiles 0–0) for biopsies from left colonic flexure, sigmoid colon and rectum. Biopsy score from the patients were median 0 (25%-75% percentiles 0–1) from left colonic flexure and median 5 (25%-75% percentiles 4–5) from rectum.

### Sample preparation and gel electrophoresis

Prior to 2-DGE whole mucosal biopsies were homogenized and lysed in buffer (1 mg biopsy/10 μl lysis buffer) consisting of 7 M urea, 2 M thiourea, 1.5% (wt/vol) pharmalyte (pH 3–10, GE Healthcare, Uppsala, Sweden), 0.8% (wt/vol) 3-[(3-chol-amidpropyl) dimethylammonium]-1-propansulfonate, CHAPS (Applichem, Darmstadt, Germany) and 1% (wt/vol) dithioerythritol in water. After 2 h incubation at room temperature, lysed cells were centrifuged for 20 min at 10,000 × *g* at 4°C, and the supernatant was aspirated. Total protein content was determined by BCA protein assay (BioRad), and 100 μg extracted protein from individual biopsies were subsequently separated using 2-DGE with proteins initially being separated in the first dimension according to isoelectric focusing using immobilized pH-gradient IPG strips (pH 5 to 8, 11 cm, BioRad, Hercules, CA). In order to achieve optimal focusing and gradient flow, the running conditions were 5 h at 200 V, 3 h at 500 V and 16 h at 3500 V. Hereafter, 12.5% sodium dodecyl sulphate polyacrylamide (SDS-PAGE) Criterion gels (BioRad) were used for second dimension separation with proteins running for 1 h at 200 V. For visualization of protein spots analytical gels were stained with Flamingo Pink (BioRad), and scanned at appropriate wavelength for fluorescence images (FX Pro Fluorescent Scanner, BioRad).

### Image analysis

Gel images were processed and gel spots detected and quantified with Progenesis SameSpot (version 3.3, Nonlinear dynamics, Newcastle, UK). Initially a few anchor spots were manually defined and followed by the build-in automated alignment procedure. Spot border lines were created from a selected reference gel and applied to all gels. After background subtraction, a gel to gel normalization based on a logarithmic abundance ratio of the spot volumes was performed in order to minimize bias from e.g. pipetting errors, when loading sample, or inconsistency in the transfer of proteins from the first to the second dimension. The output dataset contained no missing values since all spot areas were present, and quantified in all gels. The resulting normalized spot volumes were subsequently analyzed in order to identify spots with different protein expression between groups.

### In-gel digestion and peptide mass fingerprinting

For peptide mass fingerprinting (PMF) proteins of interest were subjected to in-gel digestion by addition of trypsin using an in-gel protocol, essentially as described by Jensen et al. [30]. Custom-made chromatographic columns were used for desalting and concentration of the peptide mixture prior to MS analysis [31]. Hereafter, peptides were eluted in 0.5 μL of matrix solution (15–20 g/L of α-cyano-4-hydroxycinnamic acid; Sigma Aldrich, St. Louis, USA, in 70% acetonitrile) directly onto the MALDI target plate (Bruker Daltonics GmbH, Bremen, Germany). Mass spectra were obtained using an Ultraflex MALDI-TOF tandem mass spectrometer in reflection mode (Bruker Daltonics, Bremen, Germany). A peptide calibration standard ranging from 1046.54 to 3147.47 g/mol was spotted separately onto the MALDI target plate. The ion-accelerating voltage was 25 kV and the laser frequency of 50 Hz with 200 laser spots accumulated for each spectrum. For tandem mass spectrometry (MS/MS, lift mode), the ion accelerating voltage was 19 kV, and the protein was identified based on the Mascot scores of the peptide subjected to MS/MS analysis.

### Data analysis

In order to identify spots with significantly different protein levels in the inflamed versus non-flamed mucosa of UC patients, we applied a one-way analysis of variance (*t*-test) using the logarithm of the normalized spot volumes. A false discovery rate (FDR), the proportion of significant features that turn out to be false due to multiple testing was calculated as *q*-value [32]. We also applied multivariate statistics, which is ideal for 2-DGE datasets typically consisting of long and lean data with relatively few observations (samples) and many variables (protein spots). The normalized spot volumes were imported into SIMCA 9.0 (Umetrics), and preprocessed with mean centering. For principal component analysis (PCA) of proteomic data from control persons, data was autoscaled, while group scaling was considered to be a more optimal scaling approach for PCA of proteomic data from patients. While autoscaling is based on overall standard deviation, group scaling is based upon the within-group standard deviation, and therefore gives a higher weight to the group-dependent proteins [33].

PMF mass searches were conducted in the Mascot ions search engine (Matrix Science, Boston, USA) using the Swiss-Prot database (Swiss Institute of Bioinformatics, Geneva, Switzerland). The Mascot software uses a scoring algorithm to identify the closest match and significant protein identification. The ions score is −10*Log(P), where P is the probability, that the observed match is a random event. In this study a protein score having a significance level of P < 0.05 indicated identity or extensive homology. For MS/MS identification, fragmentation of the parent ion was followed by mass searches in the database.

### Ethical considerations

All subjects received written and oral information and gave written informed consent. The study was done in accordance with the Declaration of Helsinki and was approved by the Danish Regional Ethics Committee (VN 20060041).

## Results

### Proteomic profiles of control persons and UC patients

**Figure 1:**
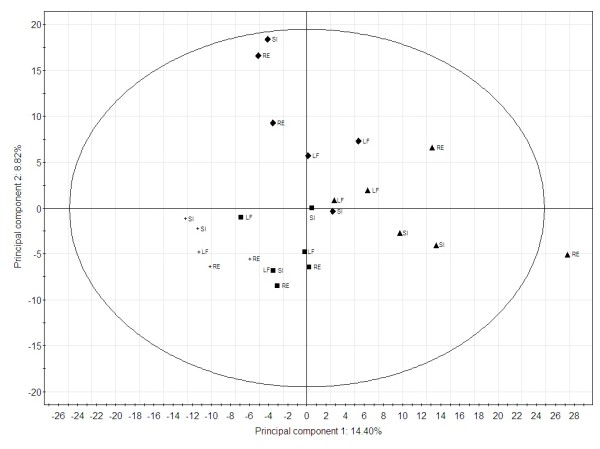
**Principal component analysis of the proteomic profiles of healthy control persons.** Results are based on 2-DGE spot volumes. Replicate biopsies were analysed from rectum (RE), sigmoid colon (SI) and left colonic flexure (LF). Control person 1 (+), control person 2 (■), control person 3 (♦) and control person 4 (▴).

The initial proteomic survey carried out on control persons indicated that the individual protein level between different locations in normal colon mucosa was not prominent, since the PCA score plot of proteomic data from control persons demonstrated a grouping according to individuals and not to specific colon positions (Figure 1). The systematic variation was more related to individual variation, than to variation between colon positions, though the first two components explain only 23% of the variation in the dataset.

The protein composition of biopsies from UC patients differed in the non-inflamed versus inflamed mucosa. After manual gel inspection of spot alignment, the image analysis annotated a total of 597 spots. Statistical significance (P < 0.05) was achieved for 222 spots being differentially expressed in inflamed versus non-inflamed mucosa, and of these 39 spots were highly significant (P < 0.0001). After correction for multiple testing by FDR, the proportion of significant spots estimated to be false positives were 4.1% for a statistical significance level of 0.05 (q = 0.041) and 0.05% for a statistical significance level of 0.0001 (q = 0.0005).

**Figure 2:**
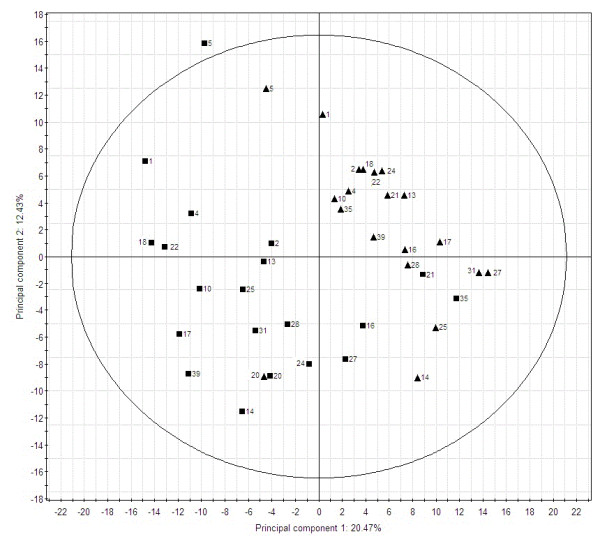
**Principal component analysis of the proteomic profiles of UC patients.** The results are based on results from 2-DGE spot volumes. Biopsies were from rectum (RE) and left colonic flexure (LF). LF (▴) and RE (■).

PCA of the proteomic profiles of biopsies from UC patients grouped control samples from left colonic flexure separately and away from inflamed rectum tissue, with the two first components explaining 33% of the variation (Figure 2). In spite of this apparent grouping of specific mucosa, it was also evident that some of the inflamed patient biopsies did not seem to exhibit an inflamed profile (RE biopsy from patient 21 and 35), and accordingly grouped with LF biopsies. Moreover, the LF biopsy from patient 20 resembled that of RE biopsies more than LF biopsies. Since our histopathological examination did not support outlier status of these biopsies, we have not excluded these patients, but ascribed it to natural variation within the biological material. Furthermore, there was no apparent grouping of the ten patients treated with 5-ASA (data not shown) and the patient group was therefore evaluated as one group.

Twenty statistically significant protein spots showed more than twofold increase or decrease in protein level when comparing inflamed and non-inflamed patient tissue, and these spots were all manually dissected and trypsinated for further MS identification. In total 14 of the protein spots were identified using MALDI-TOF MS analysis (Table 1), and a more than twofold difference in protein level was thus observed for glycerol-3-phosphate-dehydrogenase (2 hits), B-cell antigen receptor complex-associated protein beta chain, annexin A6, plasma-cell induced resident endoplasmatic reticulum protein, cytoplasmic actin, alpha enolase (2 hits), L-lactate dehydrogenase B-chain, tubulin beta 5-chain hydroxymethylglutaryl CoA synthase, selenium binding protein (2 hits) and carbonic anhydrase 2. Of these spots glycerol-3-phosphate-dehydrogenase showed the highest (3.3 fold) and alpha-enolase (spot 10) the lowest level (3.7 fold) in the inflamed tissue compared to the non-inflamed tissue. All except one (B-cell antigen receptor complex-associated protein, beta chain) of the identified spots had significant Mascot scores and the statistical significance of the spots was generally strong.

**Table 1:** List of significant proteins showing more than twofold altered protein expression within UC patients

**Spot**	**Protein Name**	**SwissProt Acc. no.**	**S cov %**^**1**^	**pI**^**2**^	**Mw**^**2**^	**Fold diff.**^**3**^	** *T* -test (p)**^**4**^
Higher level in RE							
1	Glycerol-3-phosphate-dehydrogenase	P04406	6^a^	9.3	36	3.30	1.388e-005
2^5^	B-cell antigen receptor complex-associated protein beta chain	P40259	42	5.6	26	3.05	3.776e-004
3	Annexin A6	P08133	41^a^	5.3	76	2.81	1.626e-006
4	Glycerol-3-phosphate-dehydrogenase	P04406	37^a^	9.3	36	2.70	6.178e-005
5	Plasma-cell induced resident endoplasmatic reticulum protein	Q8WU39	52^a^	5.3	21	2.61	4.785e-005
6^5^	Actin cytoplasmic	P60709	42^a^	5.5	41	2.45	1.049e-006
7	Alpha enolase	P06733	35^a^	7.7	47	2.42	5,889e-005
8	L-lactate dehydrogenase B-chain	P07195	46^a^	5.7	37	2.40	4.021e-005
9	Tubulin beta 5-chain	P07437	41^a^	4.6	50	2.18	1.801e-004
Lower level in RE							
10	Alpha-enolase	P06733	44^a^	7.7	47	3.70	1.403e-008
11	Hydroxymethylglutaryl–CoA synthase	P54868	28^a^	9.2	57	2.94	2.448e-009
12	Selenium binding protein	Q13228	42^a^	5.9	53	2.50	3.770e-007
13	Carbonic anhydrase 2	P00918	60^a^	7.0	29	2.33	8.325e-007
14	Selenium binding protein	Q13228	60^a^	5.9	53	2.08	5.508e-006

^1^Percentage sequence coverage (S cov %) is followed by ^a^ if the mascot score was significant (P < 0.05). ^2^Isoelectric point (pI) and molecular weight (Mw) are presented as theoretical values. ^3^Fold difference in protein level. ^4^p-value from *t*-test. ^5^Spot location on the gel differs from the theoretical molecular mass. ^6^The protein was identified by MS/MS.

Twenty-nine additional spots were successfully identified (Table 2). Several of these had strong statistical support for a different protein level, while others were spots with high spot volumes and therefore suitable for MALDI-TOF identification. Annexin A1 (spot 35) was the only spot not having a significant Mascot score for protein identification. MS/MS was performed on two protein spots resulting in positive identification of glycerol-3-phosphate-dehydrogenase (spot 1) and serum albumin (spot 23).

**Table 2:** List of additional significant proteins identified in UC patients

**Spot**	**Protein Name**	**SwissProt Acc.no.**	**S cov %**^**1**^	**pI**^**2**^	**Mw**^**2**^	**Fold diff.**^**3**^	** *T* -test (p)**^**4**^
Higher level in RE							
15	Superoxide dismutase	P04179	61^a^	7.0	22	1.9	1.076e-007
16	Peroxiredoxin-4	Q13162	59^a^	5.8	31	1.7	1.510e-006
17	F-actin capping protein subunit alpha-1	P52907	44^a^	5.5	33	1.6	8.491e-005
18	Inorganic Pyrophosphatase	Q15181	48^a^	5.5	33	1.4	1.461e-004
19	Triosephosphate isomerase	P60174	48^a^	7.7	27	2.0	3.173e-004
20	Actin cytoplasmic	P60709	62^a^	5.2	42	1.8	5.368e-004
21	Thiosulfate sulfurtransferase	Q16762	49^a^	6.9	34	1.3	0.004
22	Serum albumin	P02768	16^a^	5.9	71	1.4	0.005
23	Serum albumin	P02768	^5a^	5.9	71	1.8	0.005
24	Proteasome activator	Q9UL46	53^a^	5.4	28	1.2	0.005
25	Serotransferrin	P02787	24^a^	7.0	79	1.4	0.014
26	F-actin capping protein subunit beta	P47756	45^a^	5.6	31	1.1	0.025
Lower level in RE							
27	Selenium binding protein	Q13228	60^a^	5.9	53	1.4	1.525e-006
28	Protein ETHE1	O95571	56^a^	6.4	28	1.4	2.911e-006
29	Enoyl-CoA hydratase	P30084	46^a^	9.4	32	1.3	7.862e-006
30	Isocitrate dehydrogenase	O75874	35^a^	6.6	47	1.7	9.730e-006
31	Elongation factor Tu	P49411	52^a^	7.9	50	1.4	2.055e-005
32	Peroxiredoxin-6	P30041	63^a^	6.0	25	1.4	8.820e-005
33	Tubulin alpha-6 chain	Q9BQE3	29^a^	4.8	50		4.100e-005
34	Carbonic anhydrase 1	P00915	46^a^	6.7	29	2.0	1.354e-004
35	Annexin A1	P04083	34	6.7	39	1.3	2.942e-004
36	Cathepsin D	P07339	33^a^	6.1	45	1.3	3.062e-004
37	Thioredoxin-dependent peroxide reductase	P30048	55^a^	8.9	28	1.3	0.001
38	Heat shock protein 70 kDa /Serum albumin	P08107/P02768	33^a^/66^a^	5.9/5.4	70/71	1.4	0.004
39	60 kDa heat shock protein	P10809	41^a^	5.6	61	1.3	0.014
40	Serum albumin	P02768	18^a^	5.9	71	1.3	0.019
41	Triosephosphate isomerase	P60174	63^a^	6.5	27	1.3	0.019
42	Serum albumin	P02768	20^a^	5.9	71	1.3	0.021
43	Alpha-enolase	P06733	42^a^	7.7	47	1.1	0.042

^1^Percentage sequence coverage (S cov %) is followed by ^a^ if the mascot score was significant (P < 0.05). ^2^Theoretical isoelectric point (pI) and theoretical molecular weight (Mw) are presented. ^3^Fold difference in protein level. ^4^p-value from *t*-test. ^5^The protein was identified by MS/MS.

### Changes in proteomic signatures in control persons versus UC patients

In order to assess whether spots showing different protein level within UC patients could be supported by information from the control persons, the identified spots were examined in RE and LF from both control persons and UC patients. In this way, protein spots that merely reflected position effects (i.e. showing the same difference in the control group) or nonsense patterns (e.g. when the control group resemble patient RE) rather than distinct inflammation profiles could be evaluated. Using this approach five of the identified spots (alpha-enolase (spot 10 and spot 43), hydroxymethylglutaryl–CoA synthase, cathepsin D and 60 kDa heat shock protein) did not qualify for candidate marker. Alpha-enolase (spot 10) and hydroxymethylglutaryl–CoA synthase both had a significantly different protein level in the control biopsies as well as in the patient biopsies when comparing RE and LF, and showed a similar pattern in both groups that could indicate that these proteins were rather position markers than disease markers. Regarding alpha-enolase (spot 43), cathepsin D and 60 kDa heat shock protein, the protein levels in the inflamed mucosa (RE) were similar to RE and LF level from control persons.

### Candidate markers and affected biological pathways

**Figure 3:**
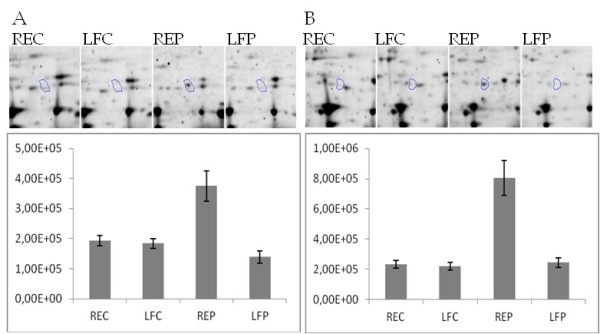
**Different protein level in rectum and left colonic flexure from control persons and UC patients.** (**A**) Glycerol-3-phosphate-dehydrogenase (spot 1) and (**B**) Glycerol-3-phosphate-dehydrogenase (spot 4). Representative 2-DGE images from the four groups and normalised protein expression of the same groups presented as mean ± standard error. Control persons (REC and LFC) and UC patients (REP and LFP).

The 43 protein spots identified was assigned to 33 individual proteins, since more than one isoform were recognized for several of the proteins. The high level of glycerol-3-phosphate-dehydrogenase (spot 1 and spot 4) in the inflamed mucosa of patients compared to non-inflamed mucosa was highly significant (Figure 3). Likewise, several isoforms of selenium binding protein were identified, but all found to have low protein levels in inflamed mucosa compared to non-inflamed tissue. In general, the proteins identified could be assigned to biological pathways such as energy metabolism, response to oxidative stress and stress-response mechanisms (Table 3).

**Table 3:** Biological processes associated with identified proteins with references to results obtained in earlier studies

**Biological process**	**Protein name**
Energy generation	triosephosphate isomerase^1,3,4^, glycerol-3-phosphate-dehydrogenase, alpha enolase^2,3^, isocitrate dehydrogenase, L-lactate dehydrogenase B-chain, inorganic pyrophosphatase, enoyl-CoA hydratase
Response to oxidative stress	selenium binding protein^1,2^, superoxide dismutase, thioredoxin-dependent peroxide reductase^1^, peroxiredoxin-1^4^, peroxiredoxin-4, peroxiredoxin-6
Stress response proteins	Alpha-enolase^3^, Hsp60^1,3^, Hsp70^1,4^

^1^Different protein level between diseased UC versus normal colon mucosa tissues using colon biopsies [22]. ^2^Different protein level between inflamed and non-inflamed tissue regions in single UC patients as identified in purified epithelial cells from biopsies [19]. ^3^Different protein level in mucosal tissue biopsies from patients with chronic refractory pouchitis before and after antibiotic/probiotic therapy [34]. ^4^Different protein level between intestinal epithelial cells from healthy subjects and CD patients [35].

## Discussion

In this study samples from both inflamed and non-inflamed tissues from each patient were analyzed. The histological assessment of the biopsies confirmed that the patient biopsy from the left colonic flexure could be assessed as non-inflamed and did not show any signs of sub-inflammation. This is the first study to assess a relatively high number of UC patients using this approach and in spite of large individual heterogeneity we found statistical support for tissue specific levels of many proteins being associated with disease exacerbation. Recently, proteomic studies have contributed with promising candidate markers being correlated with disease phenotypes, either in IBD patients in general, specifically within UC patients or between UC, CD and control persons [20-22].

In this study, the spots characterized by a larger than twofold difference in protein level were classified as the most promising markers. These spots together with the additional spots identified included proteins already known to be associated with inflammation states of IBD (e.g. triosephosphate isomerase, alpha enolase, selenium binding protein, superoxide dismutase, thioredoxin-dependent peroxide reductase, Hsp60, Hsp70) [19,22,34], as well as novel proteins (e.g. glycerol-3-phosphate-dehydrogenase, isocitrate dehydrogenase, L-lactate dehydrogenase B-chain, inorganic pyrophosphatase, enoyl-CoA hydratase, peroxiredoxin-4, peroxiredoxin-6), where the association with UC and inflammation needs replication. Integration of excised biopsies from control persons was used to strengthen our conclusions, since protein spots showing different protein levels between RE and LF colon mucosa of control persons could be excluded as merely position specific markers, and not disease markers. Furthermore, there is a strong support for regarding our significant spots as true positives since only nine false positives are expected out of 222 spots (q = 0.041), and only 0.02 spot out of the 39 highly significant (q = 0.0005).

The proteomic profile of the inflamed tissue was very distinct with 37% of the annotated protein spots having significantly different protein levels when comparing inflamed and non-inflamed patient mucosa. Focusing on the spots found to have at least more than twofold difference in protein level between patient biopsies, 70% (14 spots out of 20 possible) were successfully identified. Further identification of additional protein spots with strong statistical support strengthened the overall understanding of how inflammation changes the proteomic signature of UC patient colon biopsies. We found that, apart from the highly abundant proteins like serum albumin and cytoplasmic actin, excised spots could primarily be assigned to proteins involved in stress response mechanisms and energy metabolism. Different levels of these proteins could infer that acute inflammation in UC patients in particular impair and affect regulation of these biological processes.

Different protein profiles of several glycolytic enzymes (triosephosphate isomerase, glycerol-3-phosphate-dehydrogenase, alpha enolase) and other proteins involved in energy generation (isocitrate dehydrogenase, L-lactate dehydrogenase B-chain, inorganic pyrophosphatase, enoyl-CoA hydratase) could indicate inflammation associated alterations in energy metabolism in UC patients. This could be due to malfunction of the utilization of n-butyrate, which is the preferred fuel for colonocytes in the distal colon [36]. The observed lowered enoyl-CoA hydratase in inflamed tissue infer impaired fatty acid oxidation and the energy-deficiency is further strengthened by change in the glycolytic pathway reflected as a higher expression of glycolytic enzymes in inflamed mucosa. In accordance with the present findings, Hsieh et al. [22] observed a down-regulation of triosephosphate isomerase in UC-diseased colon mucosa, while Nanni et al. [35] observed an up-regulation of the same enzyme in intestinal epithelial cells from CD patients. In addition, a changed regulation of alpha-enolase has been associated with IBD [37], which has been suggested to reflect anaerobic glycolysis in UC patients with inflamed pouch [34]. However, changes in the level of alpha-enolase could also reflect non-glycolytic mechanisms, since the protein is also induced as a heat shock protein under hypoxic stress [38]. Induced expression of heat shock proteins due to cellular stress would probably be expected in IBD patients [39-41] and is further reflected in the lower expression of 60 and 70 kDa heat shock proteins observed in inflamed tissue.

Inflammation in IBD patients generally increases the level of reactive oxygen metabolites resulting in oxidative stress due to an imbalance between antioxidants and reactive oxygen [42,43]. Different levels of several antioxidant proteins (selenium binding protein, superoxide dismutase, thioredoxin-dependent peroxide reductase, peroxiredoxin-4 and peroxiredoxin-6) suggest an increased level of oxidative stress in UC patients, and earlier studies have specifically documented a regulation of selenium-binding protein and superperoxide dismutase in relation to patients with IBD [26,44-46]. Impaired oxidative metabolism and affected antioxidant defenses thus seem to play an important role in the pathogenesis of IBD diseases, and changed expression of involved protein is most likely related to a stimulated cell activity (e.g. neutrophils) due to severe inflammation.

Generally, the control persons demonstrated a high variability among individuals reflecting an individual protein signature rather than a strong position specific proteomic profile of biopsies excised from the same bowel location in different individuals. However, some proteins were still found to be significantly differently expressed between RE and LF tissues, indicating that these could be assigned as position markers. Two of the spots found to represent very different protein levels within the UC patients fell into this category (α-enolase (spot 10) and hydroxymethyl glutaryl-CoA synthase). These proteins could falsely have been ascribed as good candidates for disease markers if the control persons were not taken into account. Regardless of the need for control persons in terms of validation of markers as actual disease markers, and not just position markers, internal controls within the same patient seems to be a robust and more simplistic approach to implement. Here, the control persons were healthy, while in other studies, controls have been patients with e.g. colorectal carcinoma [19,45], which could complicate the interpretation of results, since UC and colorectal carcinoma are closely linked conditions, and even tumor free tissue from cancer patients could deviate from normal tissue [47]. We have not addressed the individual variation in protein level between patients, which could be substantial [19], but instead focused on protein spots having robust signatures across patients.

Overall, 2-DGE analysis has improved in relation to reproducibility, repeatability and better image analysis, and in this study the limitation related to PMF using MALDI-TOF MS technology, was due to the quantity of the protein spots and sensitivity of the instrument, which played a major role for successful protein identification. Despite its flaws, MS-based proteomic techniques are efficient in providing new insights into pathogenesis of diseases not only through identification of involved proteins but also for potential effects of different isoforms, like post-translational modifications [48]. We observed several individual proteins being assigned to multiple spots (e.g. alpha-enolase), but the underlying modifications were not studied further. Some proteins with horizontal patterns could for instance, indicate phosphorylations, changing the charge of proteins as observed in the train of protein spots assigned to selenium binding protein.

Protein extraction from whole colonic biopsies as conducted here means that a long and tedious procedure for isolation of e.g. intestinal epithelial cells is avoided. On the other hand the proteomic profile from diverse cell types of both intestinal and immune origin could potentially result in larger variability between the samples and a limited control of cell specific signatures, with a risk of some cell types overruling important expression patterns of others [8]. Essentially, whole biopsies from IBD patients contain multiple populations of cells, including inflammatory cells, and thereby proteins identified could potentially reflect other mechanisms resulting from i.e. cell death or serum. To avoid introduction of sources of errors whole biopsies is preferred for clinical use. Our study thus resembles the clinical setting in this respect. Precautions in relation to collection, processing and storage should be taken seriously for minimizing factors that potentially could alter the molecular composition of the tissue material [49]. Here, the biopsies were stored immediately on dry ice after collection and subsequently on −80°C, which in combination with gentle handlings throughout all analyses ensure a high quality of the collected material.

The patients in the UC cohort included in this study received different medical treatments, which potential could affect their protein profiles. Based on the PCA plot, the variation among the patient group treated with 5-ASA was, however, not different from the remaining patients, and therefore it was not evident that the treatment had a strong effect on the proteomic signature of the analyzed biopsies.

In conclusion, we have generated insights into the underlying mechanisms of active UC. The overall proteomic signature of inflamed colon mucosa was strong. Such assessment of biopsies from the active site of the disease reveal protein markers associated with inflamed tissue, and could be an important entry point for the discovery of new and improved non-invasive markers [24]. Thus, future studies further addressing the marker proteins found are essential for evaluation of disease specificity and clinical relevance.

## Competing interests

The authors declare that they have no competing interests.

## Authors´ contributions

NAP, VA, SP and LBL designed and performed the research; VA performed the patient recruitment, biopsy sampling, and ethical application; JCM performed the pathological evaluation of biopsies; FJ performed the data analysis; HSM conducted the 2-DGE and MS analysis; all authors contributed to the manuscript and approved the final version.

## Pre-publication history

The pre-publication history for this paper can be accessed here:

http://www.biomedcentral.com/1471-230X/12/76/prepub
